# Preferred mode of childbirth among women attending antenatal clinic at a tertiary hospital in Ghana: a cross-sectional study

**DOI:** 10.4314/ahs.v22i2.56

**Published:** 2022-06

**Authors:** Kwame Adu-Bonsaffoh, Evelyn Tamma, Joseph Seffah

**Affiliations:** 1 Department of Obstetrics and Gynaecology, University of Ghana Medical School, Accra, Ghana; 2 Holy Care Specialist Hospital, Accra, Ghana

**Keywords:** Caesarean section, mode of childbirth, Vaginal Delivery

## Abstract

**Background:**

The preference for mode of childbirth by women is emerging as a global subject of interest to many researchers, especially with the steady increase in caesarean section (CS) rates with some countries exceeding the world health organization (WHO) recommended rate. This study explored the preferences of mode of childbirth and associated factors among pregnant women in Ghana.

**Methods:**

A cross-sectional study was conducted among pregnant women at tertiary hospital in Ghana. Descriptive analysis and multivariate logistic regression were performed.

**Results:**

Among the 415 pregnant women included, 357(86.0%) and 58(14.0%) preferred vaginal delivery and CS respectively. Majority (26%) attributed their preference for vaginal delivery to its being the natural way of childbirth. The most common reason why women preferred to deliver by CS was mainly influenced by medical indication such as doctors' remarks. Significant determinants influencing preference for CS were previous childbirth [aOR:0.21, 95%CI (0.05, 0.91)], previous caesarean [aOR:20.08, 95%CI (7.73, 52.19)] and urban settlement [aOR:2.51, 95%CI (1.01, 6.29)]

**Conclusion:**

There was a clear preference for vaginal birth by pregnant women although a significant proportion preferred caesarean birth. Integration of women's preferred mode of childbirth into the clinical decision with appropriate counselling is recommended to improve women's pregnancy and childbirth experiences.

## Introduction

Women's preferences for mode of childbirth have emerged as a global subject of interest to many researchers and clinicians especially with the steady increase in the rate of caesarean sections (CS), and with some countries exceeding the World Health Organization (WHO) recommendation of 10–15%^1^. The increasing caesarean birth rates in some countries over the years do not come as a surprise since the data used in estimating this recommendation were limited mainly to northern European countries that had one of the lowest maternal and perinatal mortality^1^. Current global estimates indicate that about 18.6% of all births occurs by CS ranging from 6% to 27.2% in the least and most developed regions respectively. Latin America and the Caribbean regions have the highest rates (over 40%), followed by Northern America (32.3%), Oceania (31.1%), Europe (25%), Asia (19.2%) and Africa (7.3%). Between 1990–2014 the average global CS rate saw an increase from 6.7% to 19.1%^2^.

Over the last 20 years, debates have been ongoing by the scientific, public health and clinical communities about the dramatic increase in the use of CS globally, bearing in mind its consequences and associated risks^3^. The increasing trend in CS is partly attributed to demands by women themselves for the procedure (maternal request). These women are usually those who are highly educated and in control of making independent decisions^4^. Non-medical indications for CS such as obstetrician preference and maternal preference have been examined to explain the regional variations^5^. In the survey, 7% of Irish obstetricians prefer elective CS for themselves or partners if they were primigravida and had an uncomplicated singleton pregnancy without any clinical indication. On the other hand, 38% of the respondents preferred CS if the estimated fetal weight was 4.5 kg. Similarly, there was a consistent trend against vaginal birth if the obstetrician was female or younger^5^. Some of the reasons given by women for preferring CS include less pain, easier method, safer method, shorter labour duration among many others^6^–^9^.

In situations when complications such as severe pre-eclampsia, obstetric fistula or birth asphyxia from prolonged labour, caesarean section represents a lifesaving surgical procedure for both the mother and the unborn child^2^,^10^,^11^. However, according to WHO there is no evidence showing benefits of caesarean delivery for women or infants in the absence of a justifiable medical indication for the procedure^12^. Caesarean section, just like any other surgery, is associated with major complications such as infection, haemorrhage and uterine rupture in subsequent childbirths. Relatedly, women with limited access to comprehensive obstetric care including CS are at higher risk of adverse pregnancy outcomes^3^,^11^–^14^.

From previous literature, factors that influence the preferred mode of delivery by women include socioeconomic status, previous mode of delivery, previous childbirth experience and complications in pregnancy^2^,^4^,^15^,^16^. In Ghana, the proportion of births delivered by CS increased from 12% in 2007 to 16% in 2017. Institutional deliveries also increased from 54% in 2007 to 79% in 2017 while home deliveries declined from 45% to 20% within the same time frame^17^.

With the background of increasing trend for caesarean section globally, the objective of this study was to determine pregnant women's preference for mode of childbirth during the antenatal period and the associated determinants at a tertiary hospital in Ghana. Evaluation of the women's preferred mode of childbirth (vaginal versus caesarean) provides clinically relevant information in ensuring positive pregnancy and childbirth experience for pregnant women. Also, knowledge about women's preferences reminds clinicians of their expectations and improves shared decision making including optimal counselling during pregnancy, childbirth and postnatal periods.

## Methods

### Study design and site

This cross-sectional study was conducted at the Korle Bu Teaching Hospital (KBTH), which is the largest tertiary hospital in Ghana. The hospital is located in the capital city, Accra, and serves over 3 million people. The maternity unit of the hospital conducts approximately 10,000 childbirths annually, and the National health insurance free delivery scheme covers most of these deliveries. On daily basis, the antenatal clinic takes care about 120 pregnant and postnatal women.

This secondary analysis was part of a larger study that determined the prevalence of unintended pregnancy among women receiving antenatal care^18^. The study involved a face-to-face interview of women attending antenatal clinics based on a structured questionnaire between 1^st^ June and 31^st^ July, 2013. In this study we explored the women's preferred mode of delivery, and the reasons for their choices. Data on socio-demographic characteristics (such as maternal age, educational status, settlement (urban and urban slum/rural) and obstetric data (parity, pregnancy interval and previous caesarean) were collected. Urban slum is a highly populated residential area in the urban setting. Pregnant women attending antenatal clinic, who provided informed consent were included in the study. Women who presented in labour or with emergency complications and those who did not consent were excluded.

**Figure 1:**
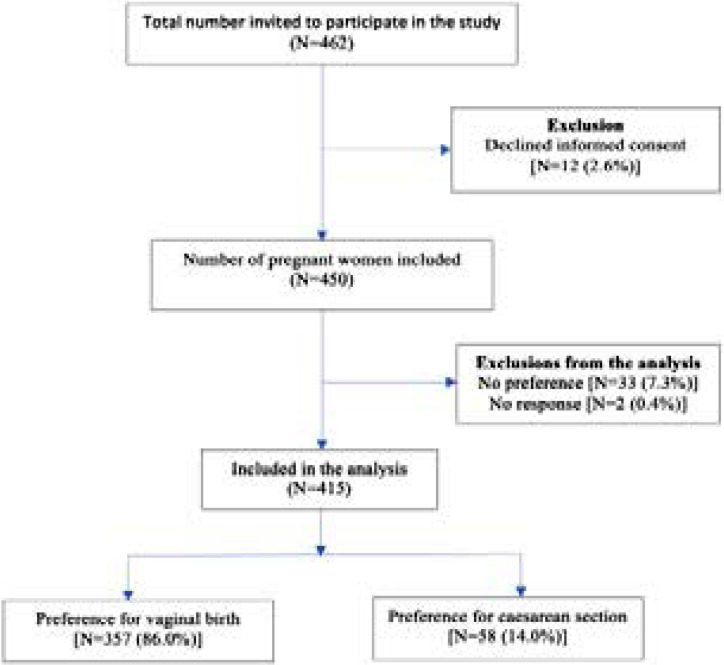
Flow chart showing recruitment of women with preference for vaginal birth and unintended caesarean section

The participants were interviewed using a structured questionnaire after they had received antenatal care services, and written provided informed consent. This approach of exit interview was necessary to enable the women to provide appropriate responses to the questions without any divided attention in waiting to see a doctor. Two well-trained female research assistants collected the data to enable the participants to feel comfortable in answering the sensitive questions. The interview lasted for about 30 minutes. The participants were informed that their participation was voluntary and opting out at any moment would not prevent them from receiving the usual quality of care from the hospital. Figure 1 indicates the flow chart for recruiting the study participants.

### Data analysis

The data were entered into Microsoft Excel spread sheet and analyzed using SPSS version 20. The outcome (dependent) variable was preferred mode of delivery, measured as a dichotomous variable (coded as vaginal and caesarean). The independent variables were pregnancy intention, maternal age, marital status, education, parity, previous caesarean, interpregnancy interval, previous stillbirth, settlement and contraceptive usage prior to index pregnancy. Preliminary descriptive analyses were performed using cross tabulations and the results presented in percentages. The bivariate logistics regression was then carried out to determine the association between the dependent and independent variables. This involved fitting one independent variable with the dependent variable to determine its effect on the dependent variable. The multivariate logistic regression was performed to determine the independent variables associated with women's preference for caesarean section. A significant result was considered at a p value of less than or equal to 0.05.

## Results

The mean ±SD maternal age of the 415 respondents included in the study was 30.6 ± 5.2 years with majority (77.6%) being 34 years and below. The nulliparous women were 128 (30.8%) and those of parity 1 and above were 287 (69.2%). Among the study participants, 119 (28.7%) and 70 (16.9%) had tertiary level and primary or no education respectively. Most of the respondents (84.6%) were married or cohabitating. The number of women with a history of previous caesarean section and stillbirth were 95 (22.9%) and 25 (6.1%) respectively. Prior to their index pregnancies, 355 (85.5%) were not using any modern contraceptives. The participants who had pregnancy interval of one year and above were 282 (91.0%) whereas 28 (9.0%) had pregnancy interval of less than one year. More than half of the respondents (63.1%) lived in urban areas and 239 (57.6%) had households consisting of 3–5 persons (Table 1).

**Table 1:** Characteristics of women with preferred mode of delivery: vaginal versus caesarean

Variable	Total	Vaginal n (%)	Caesarean n (%)	ρ value
Age				0.497
**≤ 34**	322	279 (86.6)	43 (13.4)	
**≥ 35**	93	78 (83.9)	15 (16.1)	

Parity group				0.233
Nulliparous	128	114 (89.1)	14 (10.9)	
Previous childbirth	287	243 (84.7)	44 (15.3)	
(**para ≥1**)				

Marital status				0.002
**Married/cohabitating**	351	294 (83.8)	57 (16.2)	
**Single**	64	63(98.4)	1 (1.6)	

Education				0.124
**Primary/none**	70	65 (92.9)	5 (7.1)	
**Junior high school**	114	98 (86.0)	16 (14.0)	
**Senior high school**	112	98 (87.5)	14 (12.5)	
**Tertiary**	119	96 (80.7)	23 (19.3)	

Pregnancy intention				0.606
**Intended**	270	234 (86.7)	36 (13.3)	
**Unintended**	145	123 (84.8)	22 (15.2)	

Previous caesarean				0.000
**No**	320	300 (93.8)	20 (6.2)	
**Yes**	95	57 (60.0)	38 (40.0)	

Pregnancy interval				0.022
**<12 months**	28	19 (67.9)	9 (32.1)	
**≥12 months**	282	244 (86.5)	38 (13.5)	

Prior use of contraceptives				0.516
**No**	355	307 (86.5)	48 (13.5)	
**Yes**	60	50 (83.3)	10 (16.7)	

Previous stillbirth				0.362
**No**	387	336 (86.8)	51 (13.2)	
**Yes**	25	20 (80.0)	5 (20.0)	

Settlement				0.005
**Urban**	262	216 (82.4)	46 (17.6)	
**Rural/urban slum**	153	141(92.2)	12 (7.8)	

Number of households				
**1–2**	117	104 (88.9)	13 (11.1)	0.251
**3–5**	239	206 (86.2)	33 (13.8)	
**≥ 6**	59	47 (79.7)	12 (20.3)	

### Preference for caesarean delivery

There was a significant difference (p<0.001) in the distribution of the preference of vaginal delivery and caesarean section. Among the women attending antenatal clinic, 357 (86%) prefer vaginal delivery whereas 58(14%) indicated their preference for caesarean section. The highest proportions of preference for caesarean delivery occurred among women with tertiary level of education [23 (19.3%)] and those who lived in households of six or more persons [12 (20.3%)]. The prevalence of caesarean preference was also higher among the respondents who were married or co-habiting compared to single [57 (16.2%) versus 1 (1.6%)], those with unintended compared intended pregnancies [22 (15.2%) versus 36 (13.3%)] and those who were 35 years and above compared to 34 years or less [15 (16.1%) versus 43 (13.4)]. Similarly, women with pregnancy interval of less than a year, those with a history of previous caesarean and those who lived in the urban areas had higher frequencies of caesarean preference of 32.1%, 40.0% and 17.6% respectively. Among the women with a history of previous stillbirth and those who used contraceptives before their index pregnancies, 20% and 16.7% respectively prefrred caesarean section compared to vaginal birth (Table 1).

### Determinant factors of caesarean mode of delivery

Results from the bivariate analysis shows that marital status, education, previous caesarean, pregnancy interval, and settlement were significantly associated with caesarean section. There was no significant association between a woman's pregnancy intention (intended or unintended) and the preferred mode of delivery. The multivariate logistic regression showed that parity, previous caesarean section and urban settlement were significant determinants for the preference of caesarean delivery. Women of parity 1 or more (previous childbirth) were 79% less likely to prefer caesarean delivery in their current pregnancies compared to the nulliparous women. Respondents with a previous history of caesarean section were 20 times more likely to prefer CS mode of delivery in their current pregnancies compared to those with no history of previous CS. Women residing in the urban areas were 2.5 times more likely to prefer caesarean mode of delivery compared to those residing in the urban slum (Table 2).

**Table 2:** Preference for caesarean section and its determinants among pregnant women

Variable	OR	95%CI	ρ value	AOR^a^	95%CI	ρ value
Age in years						
**≤ 34**	Ref			Ref		
**≥ 35**	1.25	0.66, 2.36	0.497	1.59	0.65, 3.91	0.309

Parity group						
**nulliparous**						
**previous childbirth (para ≥1)**	1.47	0.78, 2.80	0.235	0.21	0.05, 0.91	0.038

Marital status **Married/cohabitating**						
**Single**	Ref			Ref		
	0.08	0.01, 0.60	0.014	0.26	0.03, 2.25	0.221

Education						
**Primary/None**	Ref			Ref		
**Junior high school**	2.12	0.74, 6.08	0.161	1.66	0.44, 6.36	0.458
**Senior high school**	1.86	0.64, 5.40	0.256	2.00	0.51, 7.87	0.321
**Tertiary**	3.12	1.13, 8.61	0.029	2.65	0.66, 10.60	0.167

Pregnancy intention						
**Intended**	Ref			Ref		
**Unintended**	1.16	0.66, 2.06	0.607	1.46	0.64, 3.33	0.369

Previous caesarean						
**No**	Ref			Ref		
**Yes**	10.00	5.43, 18.42	0.000	20.08	7.73, 52.19	0.000

Pregnancy interval						
**<12 months**	Ref			Ref		
**≥12 months**	0.33	0.14, 0.78	0.012	0.48	0.14, 1.57	0.222

Prior use of contraceptives						
**No**	Ref			Ref		
**Yes**	1.65	0.59, 4.58	0.339	2.35	0.82, 6.70	0.110

Previous stillbirth						
**No**	Ref			Ref		
**Yes**	1.68	0.60, 4.66	0.324	0.78	0.19, 3.26	0.734

Settlement						
**Rural/urban slum**	Ref			Ref		
**Urban**	2.50	1.28, 4.89	0.007	2.51	1.01, 6.29	0.049

Number of households						
					
**1–2**	Ref			Ref		
**3–5**	1.28	0.65, 2.54	0.477	1.16	0.33, 4.01	0.819
**≥ 6**	2.04	0.87, 4.81	0.102	2.44	0.58, 10.30	0.227

a Adjusted for all the variables in the Table. OR: Odds ratio; AOR: adjusted odds ration; CI: confidence interval.

### Preference for vaginal delivery

**Figure 2:**
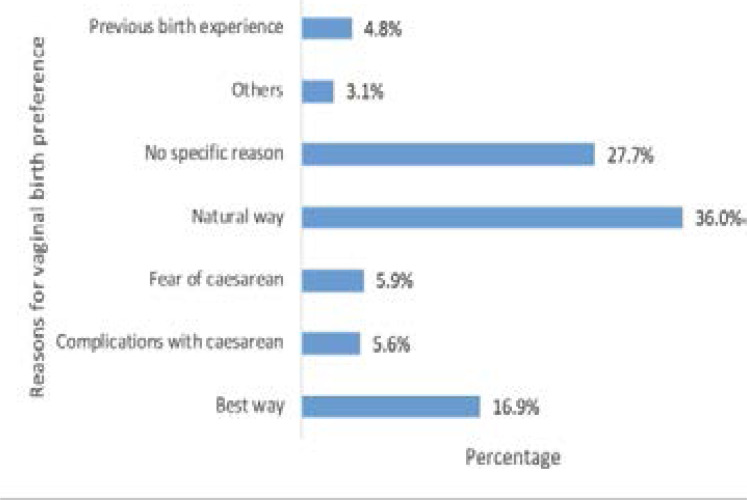
Reasons for pregnant women's preference for vaginal delivery

In all, a total of 410 women attending antenatal clinic provided reasons for their preferred mode of delivery with 354 (86.3%) in the vaginal delivery group. Among the respondents who preferred vaginal mode of childbirth, 127 (36.0%) stated that it was the natural way of giving birth, 98(27.7%) did not have any specific reason for preferring to deliver via the vaginal route and 60(16.9%) indicated that it was the best way (Figure 2). Twenty-one (5.9%) and 20 (5.6%) women preferred vaginal delivery because of the fear of caesarean section and its complications respectively.

**Figure 3:**
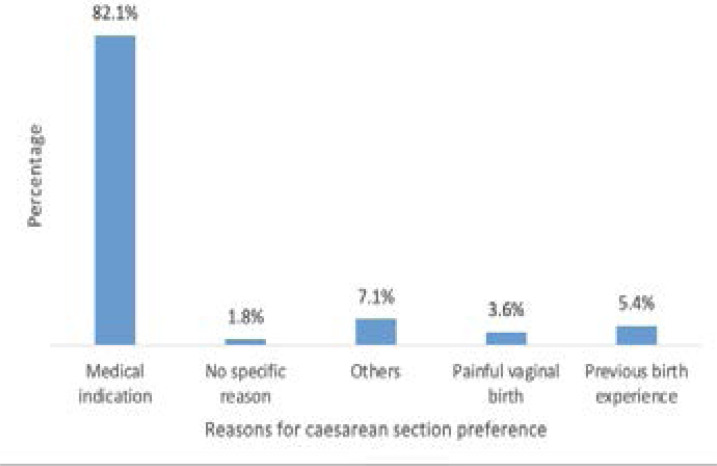
Reasons for pregnant women's preference for cesarean section

On the other hand, fifty-six (13.7%) women gave reasons for preferring caesarean mode of delivery. Forty-six respondents' reasons for preferring caesarean section consisted of medical indications (82.1%) from doctors remarks such lack of strength for vaginal delivery, prevention of complications, advanced maternal age and hypertension. The study participants whose reasons were based on previous birth experience were 3 (5.4%) and two women (3.6%) indicated that vaginal delivery was painful (Figure 3).

## Discussion

In this study, we sought to determine pregnant women's preference for the mode of childbirth and the factors associated with their preferred choices in a tertiary hospital in Ghana. Among women attending antenatal clinic there was a clear preference for vaginal delivery (86%) as against the caesarean section (14%) with a statistically significant difference. Parity (previous birth), previous caesarean and settlement (urban) were significant factors influencing the preference of caesarean section. Respondents of parity 1 or more had lower odds of preferring caesarean section as compared to the nulliparous. Women with a history of previous caesarean and those who lived in the urban area had higher odds of preferring caesarean section.

The high preference of vaginal delivery by majority of the participants in this study is consistent with other reports^6^,^8^,^19^,^20^. In a recent study in Ethiopia among postnatal women, about 87% and 13% preferred spontaneous vaginal birth and caesarean section respectively^21^. Our study population comprised pregnant women whereas Tenaw et al employed immediate postnatal women^21^, however, the preferences for mode of childbirth were similar. In a Nigerian study, 70% of pregnant women preferred vaginal delivery, 17% were indecisive and 12.5% preferred caesarean section^22^.

We determined that the odds for CS preference was reduced among women of parity 1 or more compared to nulliparous women, comparable with other studies^15^,^23^. In Ghana, Manyeh et al reported that women who have had multiple caesarean deliveries already prefer not to undergo further surgeries because of the medical risks involved in having multiple caesarean sections^15^. In another study, Bako et al determined that majority of women reported a preference for vaginal birth after previous caesarean delivery (VBAC) in Nigeria^24^. Similarly, Aslam et al also reported that women with both experiences of CS and vaginal birth in UK prefer VBAC^25^. The safety of VBAC after one CS has been supported with reliable data concerning the success rates and complications. On the other hand, women who have had vaginal delivery for their first or second delivery without any complications are more likely to prefer the same mode of delivery in their subsequent pregnancie^26^. Older nulliparous women tend to undergo CS because they are more likely to have complicated labour^27^. In this study, women with a history of previous CS had higher odds for preferring the same mode of delivery for their current pregnancies. This could be explained by the fact that previous CS increases the risk for a repeat caesarean birth in the next pregnancy. Similarly, Hildingsson et al discovered that the wish for CS was significantly associated with a previous caesarean^4^. Also, previous study carried out in Ghana also reported that previous CS was an indication for CS by majority of the respondents^28^.

Also, living in the urban settlement was significantly associated with the preference for CS. Respondents living in the urban areas had higher odds for the preference of CS compared to rural dwellers (odd ratio of 2.5). These women are more likely to be more educated and knowledgeable about the benefits and risks associated with CS^19^,^28^. Moreover, the urban dwellers tend to be financially sound to afford the increased cost associated caesarean birth. Also, living in the urban settlement also increases access to high quality medical facilities that are well equipped to safely perform caesarean section^15^. Asuquo et al reported that maternal educational status may influence women's preference for CS^22^. In this study, bivariate analysis indicated that higher educational level was significantly associated with increased odds of CS preference (unadjusted OR=3.16). However, educational status was no longer significantly associated with women's preference for CS after adjusting for confounders in the multivariable logistic regression analysis. The non-significance association between maternal education and the preference for CS might be related the small sample or selection bias especially in the tertiary public hospital setting where complicated cases are referred for specialist care. In Ghana, majority of highly educated and high social class women obtain maternity care services in renowned private hospitals, and this may have distorted the denominator factor. In a study in Turkey, maternal educational status and occupation were not associated with caesarean preference but maternal age, parity and monthly income significantly influenced the choice for caesarean^26^.

Majority of the respondents in this study attributed their preference for vaginal delivery to being the natural way to deliver. This is comparable to other studies carried out in Ghana, Turkey and Nigeria^8^,^20^,^25^. There is so much honour associated with a woman's ability to deliver by the natural means in some communities. Women who deliver by CS in these communities are considered as failures, stigmatized or mocked since CS is not considered as part of their custom^29^. About onethird of the respondents who preferred vaginal mode of delivery did not have any specific reason for wanting this choice. This could be due to lack of enough information about the mode of childbirth to make an informed choice. This calls for a further investigation regarding women's preferred choices in reproductive health and their involvement in shared decision making.

In this study, the most common reason why women preferred to deliver by CS was mainly influenced by medical indication from doctor's remarks. In a related study in Nigeria, Asuquo et al reported that about 40% of women's preference for CS was influenced by doctors' advice^22^. We also found that 1.8% of women indicated their preference for CS without any specific reason indicative of maternal request. The emergence of maternal request for CS without clinically justifiable indication is a major controversy in contemporary clinical practice, requiring further investigation.

## Strengths and limitations

The main strength of this study relates to the prospective data collection as opposed to retrospective design. Also, the inclusion of pregnant women, instead of postnatal mothers, is considered a strength of the study as pregnant women reported the true expectations for their mode of delivery in their index pregnancies. Also, this study was undertaken is the largest tertiary hospital in Ghana (metropolitan area) with pregnant women population of different ethnic and cultural backgrounds. Our study has some limitations. Firstly, information on the mode of delivery during the previous birth(s) of the participants should have been included as a predictor in the study. The preceding mode of childbirth may have influenced the current preferred delivery option. We acknowledge that the use of qualitative study design would have provided an in-depth understanding about the factors influence women's choices or preferences for childbirth options. Also, the opinions of the male partners concerning the preferences for mode of delivery would have provided significant input since, in some communities, men constitute the main stakeholders in decision making in pregnancy. However, the scope of this cross-sectional study was limited to the pregnant women. Further research is recommended to explore the male involvement in women's preferred choices of the mode of childbirth and shared decision making in reproductive health.

## Conclusion

There was a clear preference for vaginal delivery as against caesarean delivery among pregnant women. Factors that were associated with the preference of caesarean section were previous childbirth, history of previous CS and urban settlement. Women's pregnancy intentions (intended versus unintended) did not influence her choice of preferred mode of delivery. We recommend that health care providers should be aware of pregnant women's preferences for childbirth options so that the necessary information, support and care are offered to facilitate positive pregnancy and childbirth experiences. The health professionals' awareness of women's childbirth preference may facilitate shared decision making, improved provider-client communication and respectful maternity care. Further research is recommended to investigate the factors influencing women's decisions and preferences for childbirth, especially with the gradual but progressive emergence of maternal request for caesarean section in current clinical practice

## Data Availability

The data will be made available upon request subject to data sharing agreement.

## Abbreviations

CS: Caesarean section
WHO: World Health Organization
VBAC: Vaginal birth after caesarean section
